# Microbial Degradation of Amino Acid-Containing Compounds Using the Microcystin-Degrading Bacterial Strain B-9

**DOI:** 10.3390/md16020050

**Published:** 2018-02-06

**Authors:** Haiyan Jin, Yoshiko Hiraoka, Yurie Okuma, Elisabete Hiromi Hashimoto, Miki Kurita, Andrea Roxanne J. Anas, Hitoshi Uemura, Kiyomi Tsuji, Ken-Ichi Harada

**Affiliations:** 1 Graduate School of Environmental and Human Science, Meijo University, Tempaku, Nagoya 468-8503, Japan; 2 Faculty of Pharmacy, Meijo University, Tempaku, Nagoya 468-8503, Japan; y715o.c5@gmail.com (Y.H.); g0773315@ccalumni.meijo-u.ac.jp (Y.O.); elisabete.utfpr@gmail.com (E.H.H.); g0673319@ccalumni.meijo-u.ac.jp (M.K.); anasaroj@meijo-u.ac.jp (A.R.J.A.); 3 Kanagawa Prefectural Institute of Public Health, Shimomachiya, Chigasaki, Kanagawa 253-0087, Japan; uemura.aklt@pref.kanagawa.jp (H.U.); tsuji.df7@pref.kanagawa.jp (K.T.)

**Keywords:** microcystin-degrading bacteria, mycotoxin, protease, esterase, inhibitor

## Abstract

Strain B-9, which has a 99% similarity to *Sphingosinicella microcystinivorans* strain Y2, is a Gram-negative bacterium with potential for use in the degradation of microcystin-related compounds and nodularin. We attempted to extend the application area of strain B-9 and applied it to mycotoxins produced by fungi. Among the tested mycotoxins, only ochratoxin A was completely hydrolyzed to provide the constituents ochratoxin α and l-phenylalanine, and levels of fumonisin B1 gradually decreased after 96 h. However, although drugs including antibiotics released into the aquatic environment were applied for microbial degradation using strain B-9, no degradation occurred. These results suggest that strain B-9 can only degrade amino acid-containing compounds. As expected, the tested compounds with amide and ester bonds, such as 3,4-dimethyl hippuric acid and 4-benzyl aspartate, were readily hydrolyzed by strain B-9, although the sulfonamides remained unchanged. The ester compounds were characteristically and rapidly hydrolyzed as soon as they came into contact with strain B-9. Furthermore, the degradation of amide and ester compounds with amino acids was not inhibited by the addition of ethylenediaminetetraacetic acid (EDTA), indicating that the responsible enzyme was not MlrC. These results suggest that strain B-9 possesses an additional hydrolytic enzyme that should be designated as MlrE, as well as an esterase.

## 1. Introduction

Microcystins (MCs) are typical compounds produced by cyanobacteria, such as *Microcystis*, *Anabaena*, and *Planktothrix* [1]. They are cyclic heptapeptides showing potent hepatotoxicity and tumor-promoting activity [1]. In the environment, there are many bacteria which work to degrade such hazardous and harmful compounds. The first MC-degrading bacterium was isolated and identified as a *Sphingomonas* strain (ACM-3962) in 1994 [2]. Similar bacteria capable of degrading MC were reported by Dziga et al. [3]. As per their review [3], many MC-degrading microorganisms have been found and identified, and the corresponding genetic aspects with respect to MC degradation have been studied. However, in related published papers, no substrates other than MCs have been applied [4]. The purpose of the present study is to elucidate the inherent function and role of MC-degrading microorganisms in the aquatic environment.

Strain B-9, isolated from Lake Tsukui, Japan, exhibits degradation activity against MCs [1]. This strain belongs to the genus *Sphingosinecella* sp., and, based on the 16S rDNA sequence (GenBank accession no. AB084247), has 99% similarity to *Sphingosinecella microcystinivorans* strain Y2, a type of MC-degrading bacteria [5]. The *Sphingomonas* sp. strain ACM-3962 [2] was the first strain reported to degrade MCs. The cloning and molecular characterization of four genes from strain ACM-3962 revealed the presence of three hydrolytic enzymes (MlrA, MlrB, and MlrC), together with a putative oligopeptide transporter (MlrD) [6,7]. The three hydrolytic enzymes were putatively characterized as metalloproteases (MlrA and MlrC) or serine proteases (MlrB). The microcystinase MlrA catalyzes the initial ring opening of microcystin-LR (MC-LR) at the (*2S*,*3S*,*8S*,*9S*)-3-amino-9-methoxy-2,6,8-trimethyl-10-phenyldeca-4(*E*),6(*E*)-dienoic acid (Adda)-Arg peptide bond to give linearized MC-LR (Adda-Glu-Mdha-Ala-Leu-β-MeAsp-Arg). This further degrades to Adda-Glu-Mdha-Ala by MlrB, and the third enzyme, MlrC, hydrolyzes the tetrapeptide into smaller peptides and amino acids [6,7,8]. In terms of advancements, recombinant MlrA and MlrC have been prepared, and the degradation scheme has been almost completely verified [9,10]. The use of typical protease inhibitors, such as ethylenediaminetetraacetic acid (EDTA) and 1,10-phenanthroline, results in the accumulation of linear MC-LR and the tetrapeptide, which allows for the classification of the enzymes MlrA and MlrC as metalloproteases, [6,7]. Meanwhile, phenylmethylsulfonyl fluoride (PMSF) characterizes MlrB as a possible serine protease [6,7].

We extended the area of application of strain B-9 for bioremediation and applied it to the secondary fungal metabolites of mycotoxins that may have mutagenic, carcinogenic, cytotoxic, and endocrine-disrupting effects. These substances frequently contaminate agricultural commodities despite efforts for prevention, so successful detoxification methods are needed. The application of microorganisms to degrade mycotoxins is a possible strategy that shows potential for example in food and feed processing [11]; in antibiotics used in human and veterinary medicine (which can enter the environment via wastewater treatment plant effluents, hospitals, and processing plant effluents); in the application of agricultural waste and biosolids to fields; and in the case of leakage from waste-storage containers and landfills [12,13]. Such antibiotic pollution may facilitate the development and spread of antibiotic resistance [4].

Strain B-9 degrades MC-LR, the most toxic of the MCs, within 24 h [14,15]. After the discovery of strain B-9, we advanced our research on the degradation of the following compounds: non-toxic cyanobacterial cyclic peptides that are structurally different from MCs [16]; representative cyclic peptides (antibiotics) produced by bacteria [17]; physiologically-active cardiovascular and neuropeptides [18]; and the glucagon/vasoactive intestinal polypeptide (VIP) family of peptides [19]. The aforementioned experiments [17,18,19] confirmed that strain B-9 could degrade the tested peptides completely. During the application of strain B-9 to remove mycotoxins and drugs released in the aquatic environment, we obtained interesting results concerning the function of this strain. The purpose of this study was to demonstrate the additional hydrolytic enzymes (such as protease and/or esterase) of strain B-9.

## 2. Results

### 2.1. Mycotoxin and Drugs with Amide, Ester, and Sulfonamide Bonds

**Figure 1:**
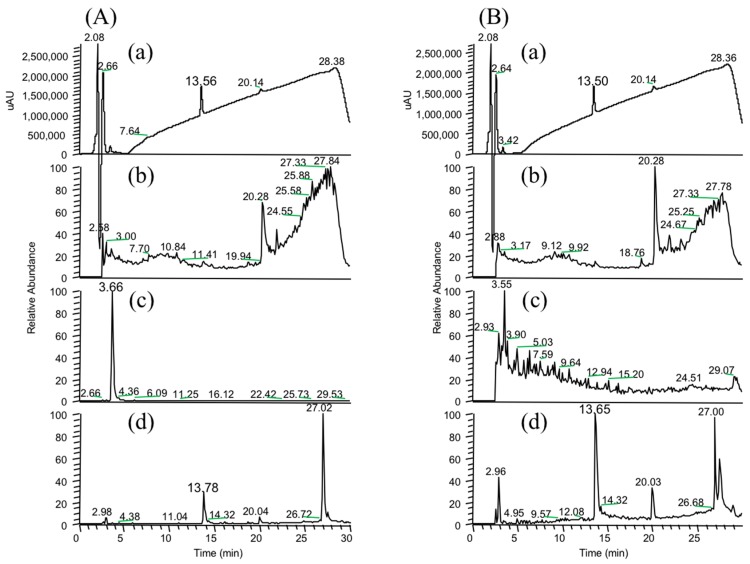
Degradation behavior of ochratoxin A at (**A**) 0 h and (**B**) 96 h after microbial degradation by strain B-9. (**a**) HPLC chromatograms; (**b**) total ion chromatograms, and selected ion monitoring (SIM) (**c**) at *m*/*z* 166.1 and (**d**) *m*/*z* 257.0 of the reaction products of ochratoxin A.

**Figure 2:**
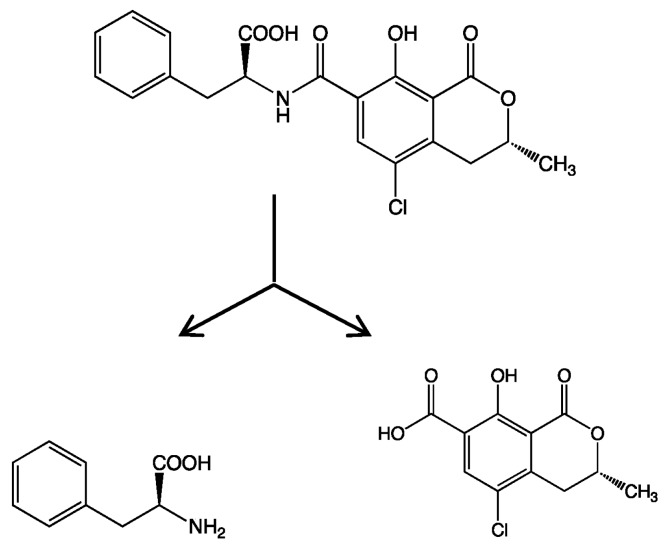
Microbial degradation of ochratoxin A (molecular weight (MW): 403.0) using strain B-9 to provide phenylalanine (MW: 165.1) and ochratoxin α (MW: 256.0).

As already mentioned [14,15], strain B-9 can degrade the phycotoxins microcystin and nodularin, as well as non-toxic cyclic peptides and linear peptides. In this study, we applied strain B-9 to mycotoxins. We monitored the degradation behavior of the five mycotoxins using HPLC and LC/MS. No degradation was observed in zearalenone, deoxynivalenol, or patulin, while the peak of ochratoxin A completely disappeared (Figure 1). The peak of fumonisin B1 reduced to a certain extent after 96 h (Figure S1). Figure 1 shows (a) HPLC chromatograms; (b) total ion chromatograms, and selected ion monitoring (SIM) (c) at *m*/*z* 166.1 and (d) at *m*/*z* 257.0 of the reaction products of ochratoxin A by microbial degradation using strain B-9 at 0 h. Although ochratoxin A appeared at 18.7 min in the HPLC chromatogram, such a peak was not observed in the data at 0 h (Figure 1A). It was found that the peak at 20.28 min was derived from strain B-9 itself by comparison of the chromatograms of ochratoxin A and a mixture of the ochratoxin A and strain B-9 broth. Consequently, the peaks at 3.66 and 13.78 min were derived from ochratoxin A, which corresponded to phenylalanine and ochratoxin α, respectively (Figure 2). After 96 h, the latter was still observed, whereas the presence of the former was significantly reduced (Figure 1B).

We tried to degrade drugs including antibiotics released in the aquatic environment using strain B-9 and selected the following drugs with amide, ester, and sulfonamide bonds: oxytetracycline (OTC), sulphaminomethoxime, sulfadimethoxime, oseltamivir, crotamiton, *N*,*N*-diethyl-*m*-toluamide, and acetaminophen. Although these were treated in the same manner as the mycotoxins, no degradation was observed (data not shown). These results suggested that strain B-9 can degrade only amino acid-containing compounds.

### 2.2. Amino Acid-Containing Compounds (Amides, Esters, and Sulfonamides)

**Figure 3:**
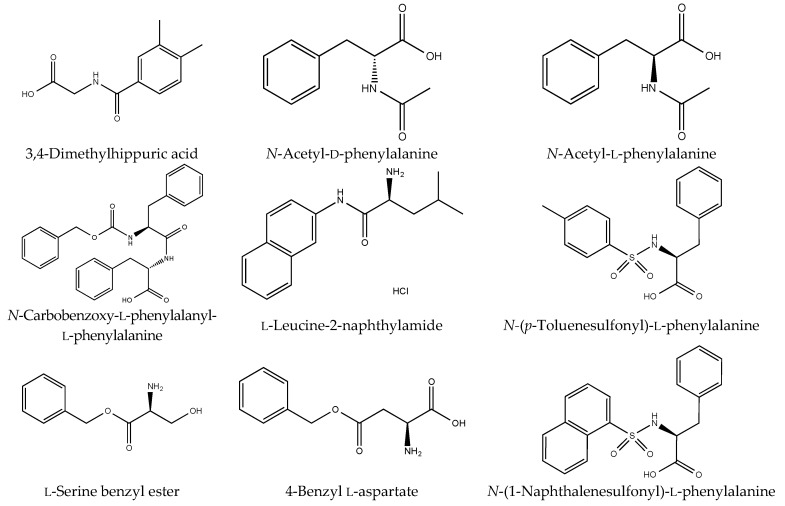
Structures of the tested amino acid-containing compounds. Amides: 3,4-dimethylhip puric acid, d- and l-*N*-acetylphenylalanines, *N*-carbobenzoxy-l-phenylalanine-l-phenylalanine, and l-leucine-2-naphthylamide; esters: serine benzyl ester, and 4-benzyl aspartate; and sulfonamides: *N*-(1-naphthalenesulfonyl)-phenylalanine, and *N*-(*p*-toluenesulfonyl)-l-phenylalanine.

**Figure 4:**
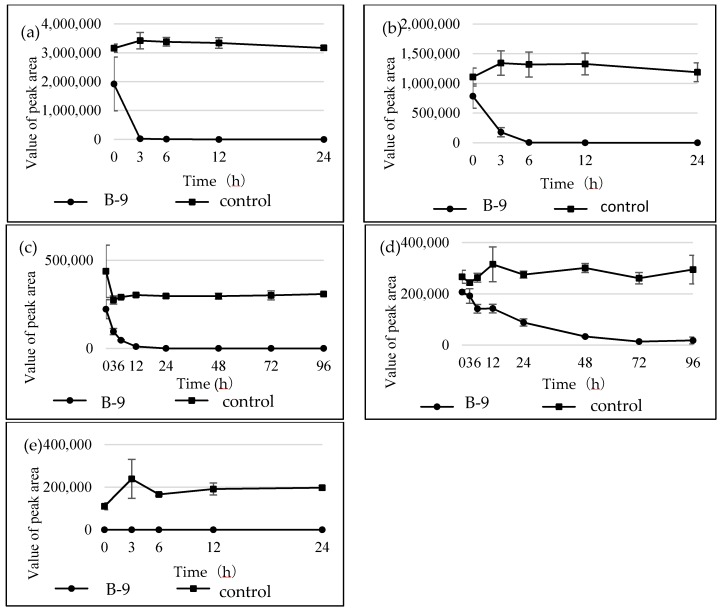
Time courses for the degradation of the tested compounds by strain B-9. (**a**) l-leucine-2-naph thylamide; (**b**) 3,4-dimethylhippuric acid; (**c**) *N*-acetyl-l-phenylalanine; (**d**) *N*-acetyl-d-phenyl alanine; and (**e**) *N*-carbobenzoxy-l-phenyl-l-phenylalanine.

**Figure 5:**
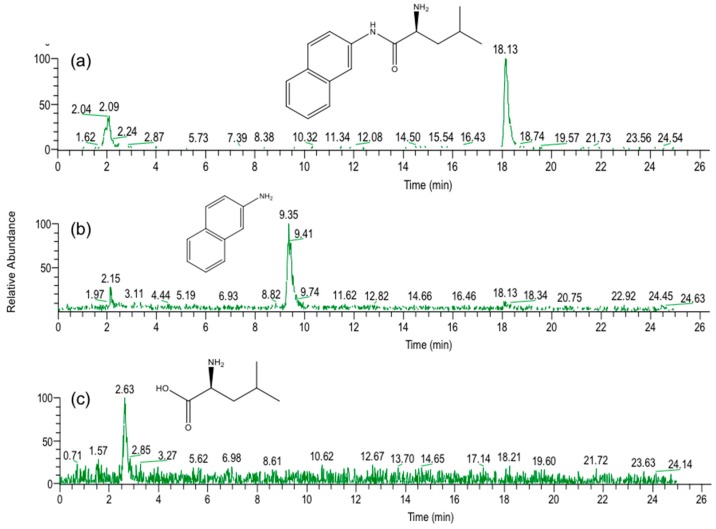
Selected ion chromatograms (SIMs) (**a**) at *m*/*z* 257.1 at 0 h; (**b**) at *m*/*z* 144.1 at 0 h; and (**c**) at *m*/*z* 132.03 at 3 h for reaction products of l-leucine-2-naphthylamide on microbial degradation using strain B-9.

The following commercially available amino acid-containing compounds with different bonds were selected. Amides: 3,4-dimethylhippuric acid, d- and l-*N*-acetylphenylalanine, *N*-carboben zoxy-l-phenylalanine-l-phenylalanine, and l-leucine-2-naphthylamide; esters: l-serine benzyl ester, and 4-benzyl l-aspartate; and sulfonamides: *N*-(*p*-toluenesulfonyl)-l-phenylalanine, and *N*-(1-naphthalenesulfonyl)-l-phenylalanine (Figure 3). l-Leucine-2-naphthylamide was treated in the same manner as the mycotoxins. The degradation proceeded smoothly and the starting material peak disappeared within 3 h (Figure 4a). A new peak was formed in the HPLC chromatogram. LC/MS showed that the starting material peak appeared after 18.13 min and the new peaks at 2.63 and 9.41 min were detected at 3 h (Figure 5). These peaks were determined to be 2-naphthylamine and leucine by selected ion chromatograms (SIM) at *m*/*z* 144.1 and *m*/*z* 132.03, respectively. The results indicated that l-leucine-2-naphthylamide was subjected to microbial degradation using strain B-9 to provide 2-naphthyl amine and leucine. As shown in Figure 4, the remaining amide compounds were also degraded and characteristic degradation behavior was observed. In addition, 3,4-dimethylhippuric acid was similarly degraded in the case of l-leucine-2-naphthylamide (Figure 4b). While *N*-acetyl-l-phenylalanine disappeared within 24 h, the d-amino acid derivative continued to appear at 96 h (Figure 4c,d). In the case of *N*-carbobenzoxy-l-phenylalanine-l-phenylalanine, the starting material peak disappeared as soon as it came into contact with strain B-9 (Figure 4e).

**Figure 6:**
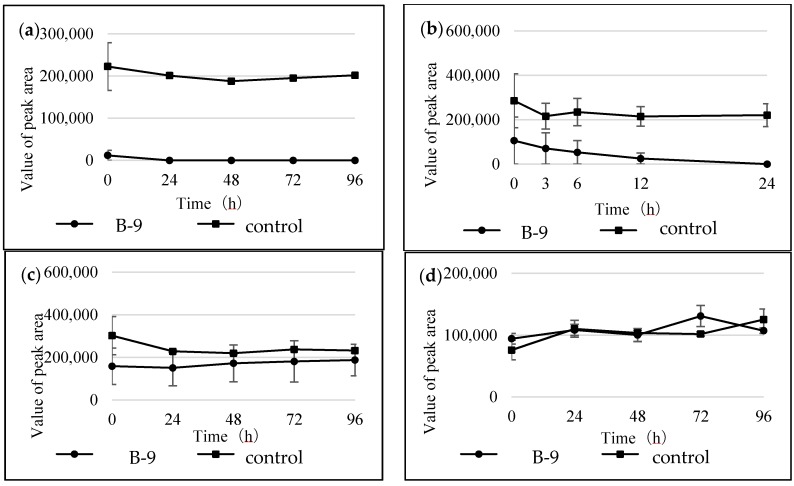
Time courses for the degradation of the tested compounds by strain B-9. (**a**) l-serine benzyl ester; (**b**) 4-benzyl l-aspartate; (**c**) *N*-(1-naphthalenesulfonyl)-l-phenylalanine; and (**d**) *N*-(*p*-toluenesulfonyl)-l-phenylalanine.

There was a common degradation behavior of the tested ester compounds, in which the starting material peaks disappeared as soon as strain B-9 came into contact with the compounds, as shown in Figure 6a,b. The resulting benzyl alcohol continued to be detected by LC/MS during the experiment (data not shown). Figure 6c,d show the degradation behavior of the sulfonamide compounds. These compounds could not be degraded by strain B-9 during the experimental period.

### 2.3. Inhibition of Hydrolysis of Amino Acid-Containing Compounds Using EDTA and PMSF

**Figure 7:**
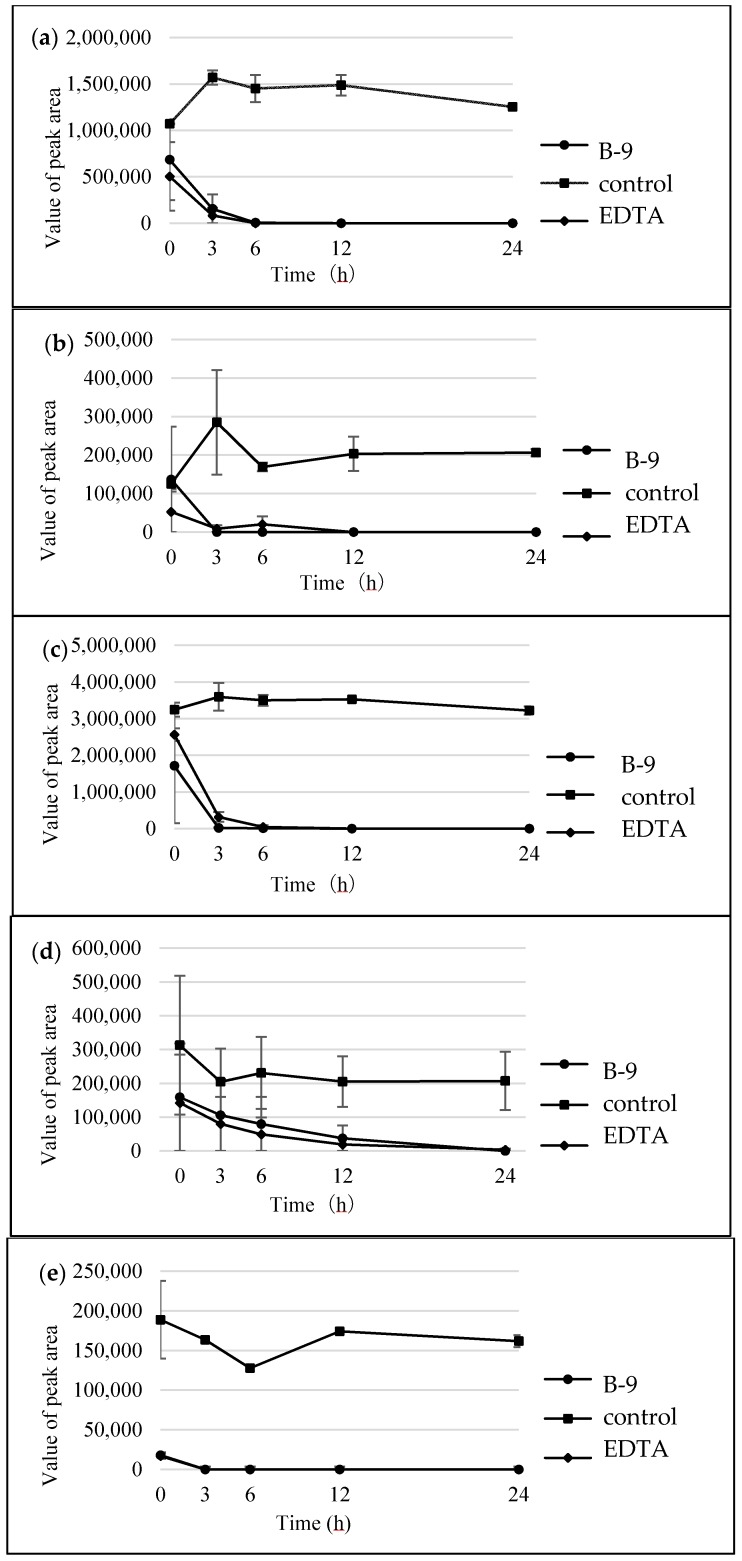
Time courses for the degradation of the tested compounds by B-9 in the presence of ethylenediaminetetraacetic acid (EDTA): (**a**) 3,4-dimethylhippuric acid; (**b**) *N*-carbobenzoxy-l-phenyl-l-phenylalanine; (**c**) l-leucine-2naphthyl amide; (**d**) 4-benzyl l-aspartate; and (**e**) l-serine benzyl ester.

When inhibitors such as EDTA or PMSF were used at 10-mM concentrations to inhibit the degradation of MCs, the microbial degradation was effectively inhibited. Consequently, the concentration was set at 10 mM in this study. To check the inhibitory activity of the prepared solution, MC-LR was subjected to the microbial degradation in the presence or absence of the inhibitor. While MC-LR (22.7 min) and the resulting tetrapeptide (20.9 min) disappeared within 24 h in the HPLC chromatogram without the inhibitor (Figure S2A), the MC-LR disappeared and the tetrapeptide continued to appear even after 72 h in the HPLC chromatogram with the inhibitor (Figure S2B). These results were consistent with our previous findings that EDTA inhibits MlrC. Figure 7 shows the time course of the degradation using EDTA and the tested compounds. These were: (a) 3,4-dimethylhippuric acid; (b) *N*-carbobenzoxy-l-phenyl-l-phenylalanine; (c) l-leucine-2-naphthylamide; (d) 4-benzyl l-aspartate; and (e) l-serine benzyl ester. They showed a common degradation behavior in that the microbial degradation of the amide and ester compounds using strain B-9 was not inhibited by EDTA. These results indicated that the degradation mentioned above was not related to MlrC. In the case of PMSF, the following compounds tested positive: 3,4-dimethylhippuric acid; l-leucine-2-naphthylamide; and 4-benzyl-l-aspartate; while other compounds tested negative: *N*-carbobenzoxy-l-phenylalanine-l-phenylalanine and a-serine benzyl ester. No definitive conclusive information was obtained.

## 3. Discussion

Strain B-9, which has 99% similarity to *Sphingosinicella microcystinivorans* strain Y2 [5], is Gram-negative and has, in several studies, shown promise for the degradation of MC-related compounds and nodularin [14,15]. Subsequently, we applied strain B-9 to other types of substrates, such as cyanobacterial peptides including depsipeptides [16], and bacterial cyclic peptides including depsipeptides [16], which are structurally different from the MCs and nodularin. Based on these results, the hydrolytic behavior using this strain is suggested as follows: (1) the reaction essentially occurs at a peptide bond in a cyclic peptide moiety to give a linearized peptide, which is more quickly hydrolyzed compared to their original ones; (2) strain B-9 primarily hydrolyzes an ester bond in a depsipeptide, in which the resulting peptides are further hydrolyzed; (3) a cyclic peptide is hydrolyzed at the acyclic part, and no further reaction occurs; and (4) the resulting linearized peptide is more quickly hydrolyzed compared to the cyclic one. In some cases, it is hard to detect the degraded peptides or amino acids due to rapid hydrolysis [16].

To confirm these observations and to further investigate the hydrolytic activities of the strain, we extended our study to physiologically active peptides such as neuropeptides and cardiovascular peptides [18]. The tested peptides were classified into two groups: (1) linear peptides, and (2) cyclic peptides with a loop formed by disulfide bond formation. The linearized peptides degraded faster than the loop-containing peptides because the loop formed by the disulfide bond was regarded as one of the degradation-resistant factors. Hydrolysis of the peptides occurred through the cleavage of various peptide bonds, and strain B-9 may bear similarities to the mammalian neutral endopeptidase (NEP) 24.11, a 94-kDa zinc metalloendoprotease widely distributed in humans and involved in the processing of peptide hormones due to its broad selectivity [20]. In a separate study, we observed the degradation behavior of the linear peptides—the glucagon/VIP family peptides (3200–5000 Da)—by strain B-9 in the absence or presence of two protease inhibitors, EDTA and PMSF. Consequently, we confirmed that one of the B-9 proteases (presumed to be MlrB), which is not inhibited by EDTA, cleaved bioactive peptides in the manner of an endopeptidase similar to NEP. Another protease, which is not inhibited by PMSF, corresponded to MlrC and cleaved the resulting middle-sized peptides to smaller peptides or amino acids [19].

In the present study, we attempted to extend the applications of strain B-9, applying it to mycotoxins produced by fungi. Among the tested mycotoxins, only ochratoxin A was completely hydrolyzed to provide the constituents ochratoxin α and l-phenylalanine (Figure 2), and fumonisin B1 levels gradually decreased to a certain extent after 96 h due to the formation of a new peak at 12.7 min (Figure S1). However, although drugs including antibiotics released into the aquatic environment were applied for microbial degradation using strain B-9, no degradation occurred. These results suggest that strain B-9 can only degrade amino acid-containing compounds. As expected, the tested compounds with amide and ester bonds were readily hydrolyzed by strain B-9, although the sulfonamides were not degraded (Figure 4 and Figure 6). In particular, the ester compounds were characteristically and rapidly hydrolyzed as soon as they came into contact with strain B-9 (Figure 6). Furthermore, the degradations of the amide and ester compounds containing amino acids were not inhibited by the addition of EDTA, suggesting that the responsible enzyme is not MlrC.

It is understood that MlrC is found in the final stage in the microbial degradation of MC, catalyzing the degradation from linearized MC and tetrapeptide to smaller peptides and amino acids. Indeed, Dziga et al. reported the role of MlrC in MC degradation, in which linearized MC and tetrapeptides could be degraded to provide Adda by the cleavage of a peptide bond between Adda and Glu by a recombinant MlrC [14]. These results suggest that strain B-9 possesses an additional hydrolytic enzyme that should be designated as MlrE. Furthermore, the results of the present study suggest that strain B-9 possesses an esterase. As mentioned above, strain B-9 degraded depsipeptides such as aeruginosins and mikamycin A, in which the cleavage at the ester bond was predominant over that of other peptide bonds [16]. However, there may be a possibility that known proteases are responsible for the ester bond cleavage.

Since their discovery in 1994, it has been believed that MC-degrading microorganisms are only responsible for MC degradation [2]. Although many reports on MC-degrading microorganisms have appeared since then [3], few papers have described their substantial function and role in the aquatic environment. As reported by our group, one of the MC-degrading microorganisms, strain B-9, is applicable to structurally diverse peptide compounds, suggesting a different function. We should investigate the detailed function of each hydrolytic and transporter enzyme, as well as a system composed of these enzymes to understand their inherent roles under aquatic conditions.

## 4. Materials and Methods

### 4.1. Chemicals

As protease inhibitors, EDTA-2Na (purity: >99.5%) and PMSF (purity: >98.5%), were purchased from Dojindo Laboratories (Kumamoto, Japan) and Sigma-Aldrich Japan (Tokyo, Japan), respectively. Acetonitrile (ACN, LC/MS grade, purity: 99.8%), methanol (MeOH, LC/MS grade, purity: 99.7%), ethanol (EtOH, special grade, purity: 99.5%), formic acid (FA, LC/MS grade, purity: 99.5%), acetic acid (AcOH, LC/MS grade, purity: 99.5%), trifluoroacetic acid (TFA, special grade, purity: 98.0%), ammonium carbonate (special grade), and 28% ammonia solution (NH_4_OH, special grade) were purchased from Wako Pure Chemical Industries, Ltd. (Osaka, Japan). Water used for the preparation of all the solutions was purified using a Milli-Q apparatus (Millipore, Billerica, MA, USA); LC/MS analysis used ultrapure water from Wako. The mycotoxins (ochratoxin A, fumonisin B1, zearalenone, deoxynivalenol, patulin) were purchased from Sigma (St. Louis, MO, USA). Drugs with amide, ester, and sulfonamide bonds and amino acid-containing compounds were obtained from the following companies: Aldrich and Sigma Japan (Tokyo, Japan), Nacalai Tesque (Kyoto, Japan), Tokyo Chemical Industry (Tokyo, Japan), and Wako Pure Chemical Industries, Ltd. (Osaka, Japan).

### 4.2. MC-Degrading Bacterium

Bacterial strain B-9, isolated from the surface water of Lake Tsukui, Kanagawa, Japan, was previously reported to degrade various MCs and nodularin [15]. This bacterium was inoculated into a flask containing 100 mL of Sakurai medium composed of peptone, yeast extract, and glucose, and incubated at 27 °C at 200 revolutions per minute (rpm) for 3 days.

### 4.3. Degradation of Tested Compounds

Two milligrams of the tested compounds was dissolved in 1 mL of EtOH and 50 μL of the solution was evaporated to dryness. One milliliter of the preincubated cell broth of strain B-9 (containing approximately 3 × 10^6^ colony forming units (CFU) mL^−1^) was added to the residue. The resulting solution was mixed, and then incubated at 27 °C for 5, 15, 30, 60, and 120 min. After incubation, 50 μL of each of these mixtures was added to 50 μL of MeOH containing 0.2% FA and filtered using an Ultrafree-MC membrane centrifuge-filtration unit (hydrophilic polytetrafluoroethylene (PTFA), 0.20 μm, Millipore, Bedford, MA, USA) to stop the degradation and to eliminate proteins. Each supernatant was then analyzed by HPLC and LC/MS.

### 4.4. Enzyme Inhibition

Enzyme inhibitors were prepared as follows: EDTA was prepared as a 200-mM stock solution in water and was used at an assay concentration of 10 mM. PMSF was prepared as a 200-mM stock solution in EtOH and was used at an assay concentration of 10 mM. The cell broth and required inhibitor were preincubated at 27 °C for 30 min.

### 4.5. High-Performance Liquid Chromatography

The degradation process was monitored by HPLC-photo diode array (PDA) at 220 or 254 nm. The system consisted of a pair of LC 10AD VP pumps, a DGU 12A degasser, a CTO 6A column oven, an SPD 10A VP photodiode array detector, and an SCL 10A VP system controller (Shimadzu, Kyoto, Japan). Five microliters of the filtered sample were loaded onto a TSK-gel Super ODS column (2.0 μm, 2.0 × 100 mm, TOSOH, Tokyo, Japan) at 40 °C. The mobile phase was 0.1% formic acid in water (A) and 0.1% formic acid in methanol (B). The gradient conditions were initially 40–90% B for 20 min, and the flow rate was 200 μL/min.

### 4.6. Liquid Chromatography/Ion Trap Mass Spectrometry

The sample, column, mobile phase, and gradient conditions were the same as those used for the HPLC analysis (12). The LC separation was performed using the Agilent 1100 HPLC system (Agilent Technologies, Palo Alto, CA, USA). Five microliters of the sample was filtrated using an Ultrafree-MC membrane centrifuge filtration unit (hydrophilic PTFE, 0.20 μm, Millipore, Bedford, MA, USA) and loaded onto a TSK-gel Super ODS column (2.0 μm, 2.0 × 100 mm, TOSOH, Tokyo, Japan) at 40 °C. The mobile phase was 0.1% formic acid in water (A) and 0.1% formic acid in acetonitrile (B). The flow rate was 200 μL/min with UV detection at 254 nm. The gradient conditions were initially 10–90% B for 40 min. The entire eluate was directed into the mass spectrometer, where it was diverted to waste 2.5 min after injection to avoid any introduction of salts into the ion source. The MS analysis was accomplished using a Finnigan LCQ Deca XP plus ITMS (Thermo Fischer Scientific, San Jose, CA, USA) equipped with an electrospray ionization (ESI) interface. The ESI conditions in the positive ion mode were as follows: capillary temperature 300 °C, sheath gas flow rate 35 (arbitrary unit), ESI source voltage 5000 V, capillary voltage 43 V, and tube lens offset 15 V. Various scan ranges were used according to the molecular weights of the tested compounds.

## Supplementary Materials

The following are available online at http://www.mdpi.com/1660-3397/16/2/50/s1, Figure S1: Total ion chromatograms (a) and (b) and selected ion monitoring (SIM) at *m*/*z* 722.4 (c) and *m*/*z* 564.3 (d) of fumonisin B1 and a reaction product by microbial degradation using B-9 at 0 h (A) and 96 h (B), respectively, Figure S2: (A) HPLC chromatograms of MC-LR by B-9 without EDTA after 0, 6 and 24 h. (B) HPLC chromatograms of MC-LR by B-9 with EDTA after 0, 6 and 72 h.
