# Supplementary material for: Microbial Degradation of Amino Acid-Containing Compounds Using the Microcystin-Degrading Bacterial Strain B-9
Source: Mar Drugs. 2018 Feb 6;16(2):50. doi: 10.3390/md16020050 (PMC5852478; doi:10.3390/md16020050)
Supplement: Supplementary file 1 [file marinedrugs-16-00050-s001.ppt]

## Slide 1
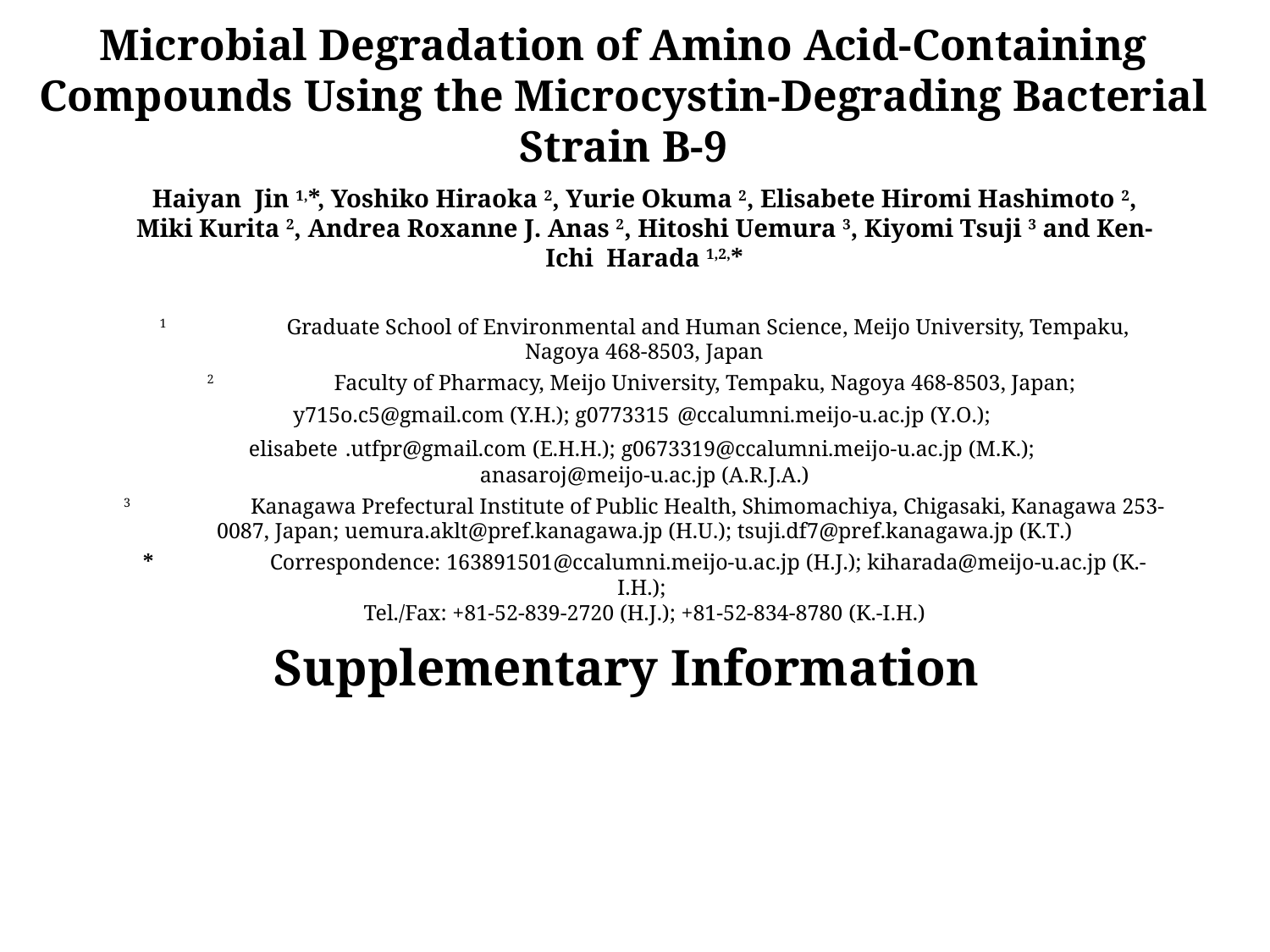

# Microbial Degradation of Amino Acid-Containing Compounds Using the Microcystin-Degrading Bacterial Strain B-9
Haiyan  Jin 1,*, Yoshiko Hiraoka 2, Yurie Okuma 2, Elisabete Hiromi Hashimoto 2, Miki Kurita 2, Andrea Roxanne J. Anas 2, Hitoshi Uemura 3, Kiyomi Tsuji 3 and Ken-Ichi  Harada 1,2,*
1	Graduate School of Environmental and Human Science, Meijo University, Tempaku, Nagoya 468-8503, Japan
2	Faculty of Pharmacy, Meijo University, Tempaku, Nagoya 468-8503, Japan;
y715o.c5@gmail.com (Y.H.); g0773315 @ccalumni.meijo-u.ac.jp (Y.O.);
elisabete .utfpr@gmail.com (E.H.H.); g0673319@ccalumni.meijo-u.ac.jp (M.K.);
anasaroj@meijo-u.ac.jp (A.R.J.A.)
3	Kanagawa Prefectural Institute of Public Health, Shimomachiya, Chigasaki, Kanagawa 253-0087, Japan; uemura.aklt@pref.kanagawa.jp (H.U.); tsuji.df7@pref.kanagawa.jp (K.T.)
*	Correspondence: 163891501@ccalumni.meijo-u.ac.jp (H.J.); kiharada@meijo-u.ac.jp (K.-I.H.);
Tel./Fax: +81-52-839-2720 (H.J.); +81-52-834-8780 (K.-I.H.)
 Supplementary Information

## Slide 2
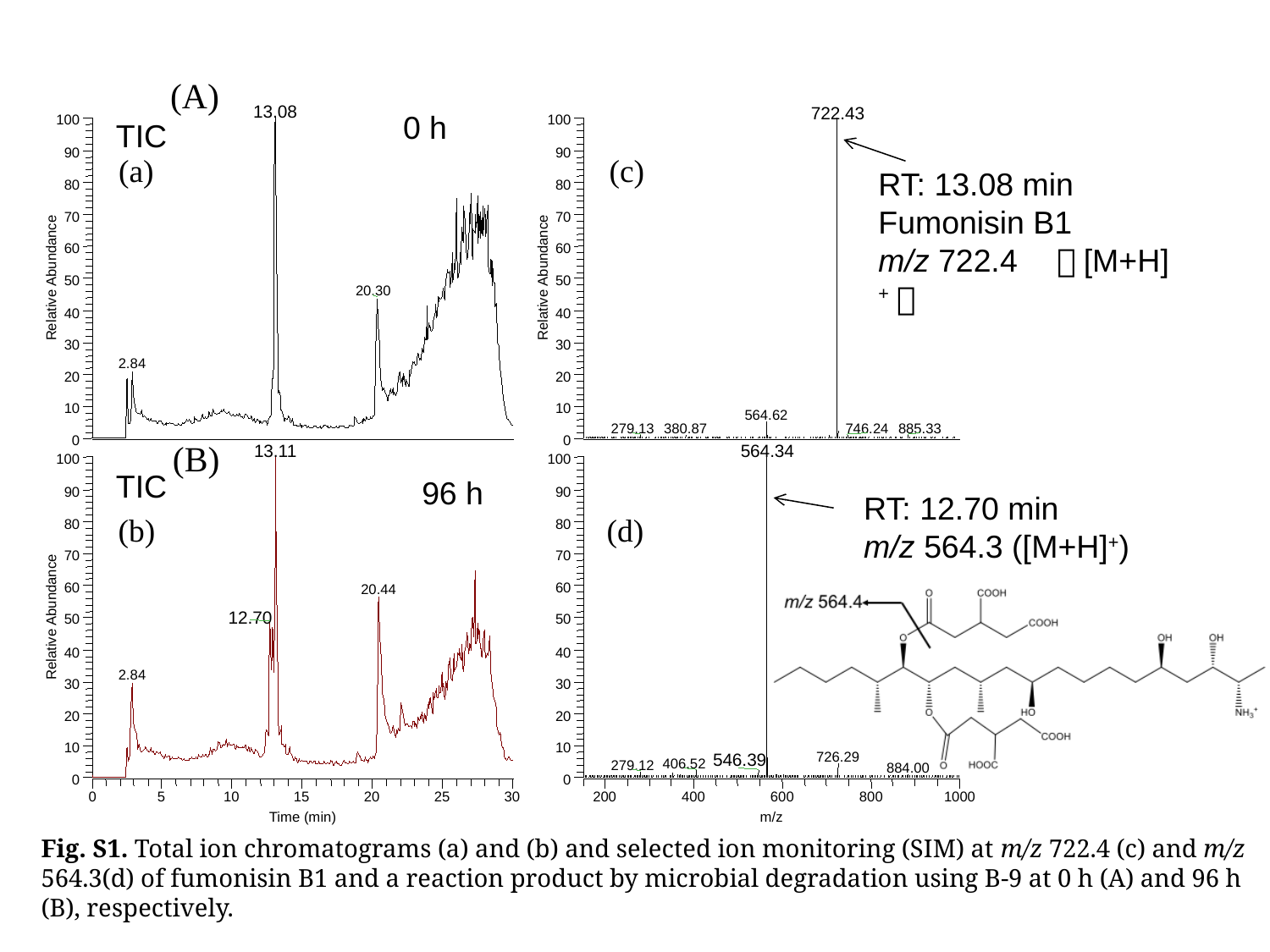

(A)
13.08
0 h
722.43
TIC
100
100
90
90
RT: 13.08 min
Fumonisin B1
m/z 722.4　（[M+H]+）
80
80
70
70
60
60
Relative Abundance
Relative Abundance
50
50
20.30
40
40
30
30
2.84
20
20
10
10
564.62
279.13
380.87
746.24
885.33
0
0
13.11
564.34
100
100
TIC
96 h
RT: 12.70 min
m/z 564.3 ([M+H]+)
90
90
80
80
70
70
60
60
20.44
12.70
Relative Abundance
50
50
40
40
2.84
30
30
20
20
10
10
546.39
726.29
406.52
279.12
884.00
0
0
0
5
10
15
20
25
30
200
400
600
800
1000
Time (min)
m/z
(a)
(c)
(B)
(b)
(d)
Fig. S1. Total ion chromatograms (a) and (b) and selected ion monitoring (SIM) at m/z 722.4 (c) and m/z 564.3(d) of fumonisin B1 and a reaction product by microbial degradation using B-9 at 0 h (A) and 96 h (B), respectively.

## Slide 3
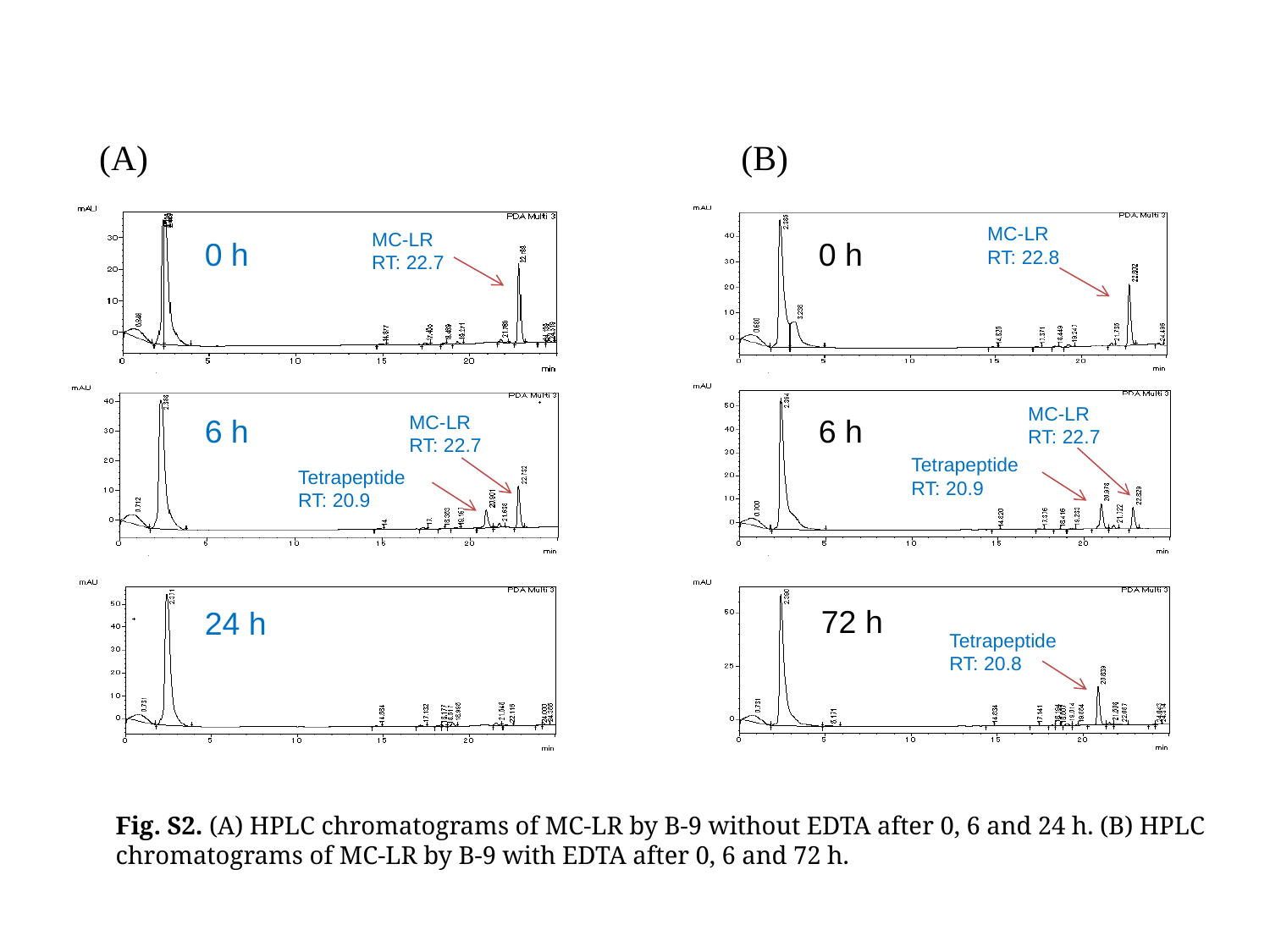

(A)
(B)
MC-LR
RT: 22.7
0 h
MC-LR
RT: 22.7
6 h
Tetrapeptide
RT: 20.9
24 h
MC-LR
RT: 22.8
0 h
MC-LR
RT: 22.7
6 h
Tetrapeptide
RT: 20.9
72 h
Tetrapeptide
RT: 20.8
Fig. S2. (A) HPLC chromatograms of MC-LR by B-9 without EDTA after 0, 6 and 24 h. (B) HPLC chromatograms of MC-LR by B-9 with EDTA after 0, 6 and 72 h.
